# Optimization and DFT study for boosted electooxidation of formic acid at NiOx modified Pt using urea derivatives as blending fuels

**DOI:** 10.1038/s41598-024-84492-z

**Published:** 2025-01-18

**Authors:** Aya M. Saada, Mohamed E. Ghaith, Ahmed A. El-Sherif, Mohamed S. El-Deab

**Affiliations:** 1 Department of Biochemical Engineering, Faculty of Energy and Environmental Engineering (FEEE), The British University in Egypt (BUE), El-Shorouk, Cairo 11837 Egypt; 2 Department of Chemistry, Faculty of Science, Cairo University, Cairo, Egypt

**Keywords:** Electrocatalysis, Fuel utilization, Fuel blends, Nanoparticles, DFT calculations, Energy science and technology, Materials science, Nanoscience and technology

## Abstract

This paper addresses the enhancement of formic acid electrooxidation (FAO) at Pt and Pt-NiOx nanoparticles based-catalysts assisted with urea derivatives as blending fuels. Blending formic acid with various ratios of urea derivatives showed noticeable enhancements of FAO as demonstrated by a favorable negative shift of its onset potential (*E*_onset_) and increase of its peak current density concurrently with suppression of the amount of CO poisoning reaction intermediate. Among all the used derivatives, phenyl urea (PU) showed superior enhancing effect towards the direct FAO with a minimal CO formation together with a favorable negative shift of *E*_onset_ by 150 mV. The superb enhancing effect of PU over U and/or other urea derivatives (investigated herein) is attributed mainly to the withdrawing inductive effect of the phenyl group attached to urea. That is the formation of 8 membered ring via hydrogen bonding between PU and formate anion is thought to enrich the electrode/electrolyte interface by FA in such a favorable orientation facilitating the C-H scissoring resulting in the direct oxidation of FA (to CO_2_) with almost no possibility for CO formation. DFT calculations are used to support this assumption in line with experimental results.

## Introduction

Fossil fuels (petroleum, natural gas and coal) represent the major source of world-wide energy supply for developed and developing countries. However, it is non-renewable and not an ecofriendly way to produce energy, also polluting the air by rising the global temperatures due to greenhouse gases emissions. As a result of the significant increase in the industrial and technological fields which need huge amount of energy and the rapid world’s population who wants to live a healthy life on the earth^1,2^, thus, researchers are focusing on the development of eco-friendly renewable energy resources such as fuel cells (FCs), solar energy, biomass, wind turbines, geothermal as well as hydropower resources with a target of decarbonization^1–4^. FCs are viewed as a major player in the global energy mix, i.e., transition towards more sustainable and low-carbon energy systems^5–7^. FCs are considered as a unique entry into the era of quiet, noiseless, and eco-friendly electrical energy production devices. This technology is highly versatile and can be used to power variety of applications including transportation, stationary power generation, and portable devices. Recently, because of its comparatively high energy density, high efficiency, variable size, minimal environmental impact, and low operating temperature than other renewable energy sources, FCs have drawn much attention for being potent energy devices for many applications and became capacitive energy source, principally in transportation, e.g., electric vehicles and space crafts, portable or stationary electronic devices^8,9^. So far, direct liquid fuel cells (DLFCs) have received significant attention as a promising renewable way of energy production attributing to their unique features over the traditional hydrogen FCs, including high energy density, easy handling fuel (as liquid), transport, and storage^5–7^. In DLFCs small organic molecules were intensively investigated as a liquid fuel^8–11^. One of these FCs is the direct formic acid fuel cell (DFAFC) it is quite used due to its theoretical open circuit potential of 1.45 V, provides an energy density of 1.4 kWh/kg, and a very low crossover rate^12–15^.

It is agreed upon in DFAFC research that formic acid (FA) oxidation (FAO) at Pt catalysts follows a dual path mechanism depending on the crystallographic orientation of the Pt surface^13,14^. The first pathway is the dehydrogenation pathway, in which FA is directly oxidized to CO_2_ according to:

1 $${\text{HCOOH}} \to {\text{CO}}_{2} + 2{\text{H}}^{ + } + 2{\text{e}}^{ - }$$

whereas, the other pathway is the dehydration, in which FA is decomposed (at the Pt surface) to H_2_O, and the poisonous CO, then the latter is further oxidized to CO_2_ according to^14–20^:

2 $${\text{HCOOH}} \to {\text{CO}}_{{({\text{ads}})}} + {\text{ H}}_{{\text{2}}} {\text{O}} \to {\text{CO}}_{{\text{2}}} + {\text{2H}}^{ + } + {\text{2e}}^{ - }$$

However, it is still suffering from drawbacks including poor stability due to the blocking of the Pt active surface site by the exhaustive adsorption of CO, which leads to deterioration of its catalytic activity^1,5^.

**Fig. 1:**
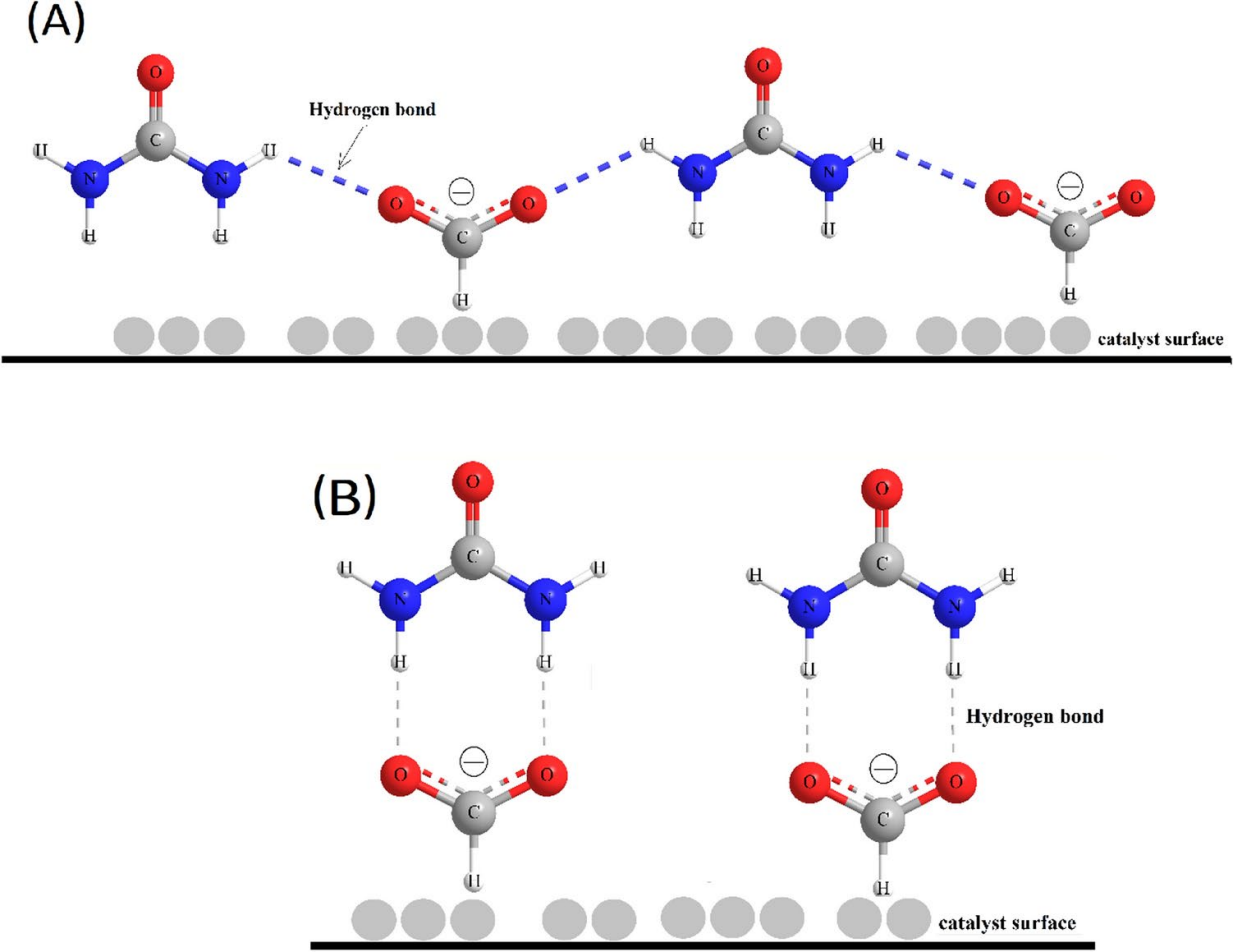
Schematic illustrations of the interaction of FA and urea (via H-bonding) in the vicinity of the catalyst surface. (**A**) expanding polymeric linear chain and (**B**) cyclic-ring structure. Atom color code: (C: grey, N: blue, O: red, H: white).

Recently, a new strategy is introduced to enhance the oxidation of small organic molecules like FA and ethylene glycol (EG) at the anodes of FCs via the addition of small fractions of another organic molecule^10,20–22^ in order to increase the fuel utilization by the proper adsorptivity of the reacted target molecule (to be oxidized; herein FA) at the active sites of the anodic catalyst (Pt or NiO_x_/Pt) and/or to reduce the non-desired oxidation pathway (CO formation). The proposed enhancing properties of the fuel blends originate from the tuned preferential orientation of the molecules assisted by the H-bonding formation between the two blended molecules^10,23–26^. That is blending small organic molecules (e.g., ascorbic acid (AA) and formic acid (FA)) with urea showed an enhanced electro-oxidative catalytic activity at nanoparticles-based anodes. Urea molecules are believed to serve as anchoring antennae molecules for AA and FA in the vicinity of the catalyst surface, thus, enabling a facile electron transfer by providing a favorable geometry for the adsorption of AA or FA at the catalyst surface and facilitates its oxidation^10,27^. This has been explained by the assumption of the formation of either linearly expanding chain structures or cyclic ring structures with urea through hydrogen bonding (linkage between the two -NH2 groups of urea and oxygen atoms in the small organic molecules). Figure 1 shows schematic illustrations of the two proposed interactions between FA and urea (A: represents the formation of a linearly expanding chain between urea and FA via H-bonding; and B: represents the possible formation of cyclic structure via H-bonding in the vicinity of the catalyst surface for a facile scissoring of the C-H bond)^10,27^.

This strategy opens several possibilities for the blending and the smart selection of the binary fuels. In this paper, the electro-oxidation of FA blended with urea and a series of its derivatives are studied at Pt/GC and NiOx/Pt/GC electrodes in alkaline medium with an aim to maximize the current density for the direct oxidation pathway while suppressing the poisoning CO-formation pathway. Methyl urea, dimethyl urea, tetramethyl urea and phenyl urea are tested as blending fuels (with FA) and their effect on the direct FAO is investigated. The experimental results are discussed and a plausible explanation is given assuming the formation of 8-membered ring via H-bonding between urea derivatives and FA in such a way that facilitates the direct oxidation of FA to CO_2_. Density functional theory (DFT) calculations are carried out to support the proposed enhancing role of each derivative.

## Experimental

### Materials and methods

All chemicals which used in this work were of analytical grade, which were purchased from Sigma Aldrich and Merck (Germany) and were used as received without further purification. All solutions were prepared using distilled water. Electrochemical measurements, e.g., cyclic voltammetry (CV), chronoamperometry (CA), linear sweep voltammetry (LSV), were carried out in a three-electrode two-compartment electrochemical cell. A glassy carbon (GC) electrode of (d = 3.0 mm) was used as working electrode after mechanically polished by a 3000 grit and then with increasingly finer alumina powder slurries in water (down to 0.06 µm), it is completely cleaned with fresh distilled water in a sonication bath. All measurements were performed at room temperature (25 ± 1 °C) using a Bio-Logic potentiostat (model VSP-300). A spiral Pt wire and an Ag/AgCl/KCl(sat.) were used as the counter and the reference electrodes, respectively.

### Catalyst preparation

The electrodeposition of the of Pt nanoparticles on GC substrate (nano-Pt/GC) was carried out by applying a potential step electrolysis from 1 V to 0.1 V vs. Ag/AgCl/KCl(sat) for 3 min from an acidic solution of 0.1 M H_2_SO_4_ containing 10.0 mM H_2_PtCl_6_^23^. For the preparation of the binary NiOx-Pt/GC electrode, the thus prepared nano-Pt/GC is further modified with the electrodeposition of Ni by applying a constant potential of − 1.0 V vs. Ag/AgCl/KCl(sat) in an aqueous solution of 0.75 M (NH_4_)_2_SO_4_ contains 40.0 mM NiSO_4_. Followed by activation of Ni metal to form various oxygenated species (NiOx; oxides, hydroxides, oxyhydroxides) by employing CV from 0.850 V to 0.60 V vs. Ag/AgCl/KCl(sat) at potential scan rate 50 of mV s^−1^ in 0.1 M KOH for 6 cycles^24^.

### Materials characterization of the prepared electrodes

The prepared nanostructures were probed with FE-SEM using a field-emission scanning electron microscope (FE-SEM, FEGESEM, provided by (Thermo- Scientific USA, model Quattro S) equipped with an EDS unit connected to it. XRD, (BURKER, D8 DISCOVER) operated with Cu target (λ = 1.540 Å) and scan speed = 0.05°/s was used to probe the crystallographic structure, chemical composition of the various electrodeposited catalysts. XPS was measured using Al Kα radiation to study the surface composition and oxidation states of nanoparticles. The binding energies derived from XPS measurements have been calibrated to the C 1 s spectrum (at 284.5 eV) of the GC support.

### Electrochemical measurements

The GC modified electrodes (i.e., nano-Pt/GC and nano-NiOx-Pt/GC) were electrochemically characterized by measuring the CV response in 0.1 M KOH (see Fig. 5 in the revised version) in the potential region from − 0.85 to 0.6 V vs. Ag/AgCl/KCl at a potential scan rate of 50 mV/s (c.f. Fig. 5). Whereas, the electrocatalytic performance of nano-Pt/GC and nano-NiOx/Pt/GC anodes towards formic acid electro-oxidation in alkaline medium (0.1 M KOH) containing 0.3 M formic acid (in the absence and the presence of minute amounts of urea or its derivatives) was probed by CV LSV measurements in the potential window of − 0.2 to 1 V vs. Ag/AgCl/KCl at a potential scan rate of 50 mV/s (c.f. Fig. 6).

### Computational method

A plausible model is proposed in which the formation of 1:1 binary molecular structures is assumed between FA and urea derivatives with favorable lower energy and geometry as depicted by computational calculations using Gaussian 09W software, applying density function theory (DFT) using B3LYP method/Basis Set = 6-311G^21^ (the obtained results are cited hereafter c.f. Table 1). The density functional theory (DFT) approach is used to probe the total energy and the dipole moment of the individual fuel molecules, i.e., FA in the absence and the presence of various blending additives, e.g., urea, methyl urea, dimethyl urea, phenyl urea and tetramethyl urea.

**Table 1:** Optimized structures and geometries of formate ion, U, urea derivatives and several binary systems between FA and urea derivatives with the corresponding calculated total energy (a.u.), *E*_HOMO_ (a.u.) and dipole moment (Debye).

Compound	Optimized structure	E_HOMO_ (a.u.)	Dipole moment (Debye)	Total energy (a.u.)
Formate ion		0.02095	1.950	− 189.109
Urea		− 0.24681	4.629	− 225.194
Methyl urea		− 0.23672	4.736	− 264.490
Tetramethyl urea		− 0.22188	3.811	− 382.372

Chemical compound	Optimized structure	EHOMO a.u	Dipole moment (Debye)	Total energy (Hartree)
1,1-dimethyl urea		− 0.22542	4.581	− 303.784
N,N′-dimethyl urea		− 0.23434	4.632	− 303.786
Phenyl urea		− 0.21355	4.443	− 456.192

1:1 interaction	Optimized structure	Dipole moment (Debye)	Total energy (Hartree)	
Formate ion: urea (8-memberd ring)		0.039	− 414.377	
Formate ion: methyl urea		2.895	− 453.656	
Formate ion: tetramethyl urea		4.621	− 571.520	
Formate ion: 1,1-dimethyl urea		4.425	− 492.935	

1:1 interaction	Optimized structure	Dipole moment (Debye)	Total energy (Hartree)	
Formate ion: N,N′-dimethyl urea		2.822	− 492.955	
Formate ion: phenyl urea		4.733	− 645.387	

## Results and discussion

### Morphological, structure and electrochemical characterization

**Fig. 2:**
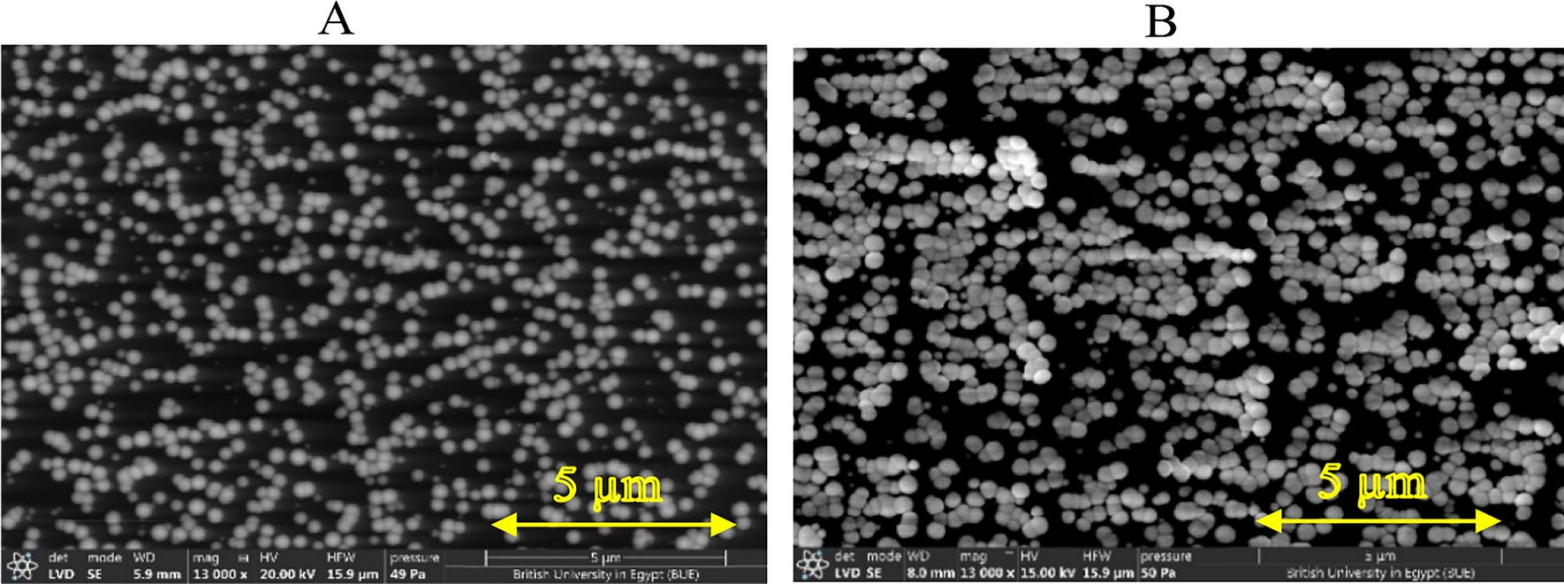
SEM image of (**A**) Pt/GC and (**B**) NiOx/Pt/GC electrodes at magnification of 13000 x.

Figure 2 shows SEM images of the papered catalysts (A) Pt/GC, and (B) NiOx/Pt/GC electrodes. Figure 2A indicates that nano-Pt with an average particle size of 200 nm are covering the GC surface. The effort is committed next to evaluate the composition and crystal structure of the electrode involved in this investigation, One the other hand Fig. 2B shows both particles of Pt and NiOx on GC surface, further confirmation will be with EDX and electrochemical analysis.

**Fig. 3:**
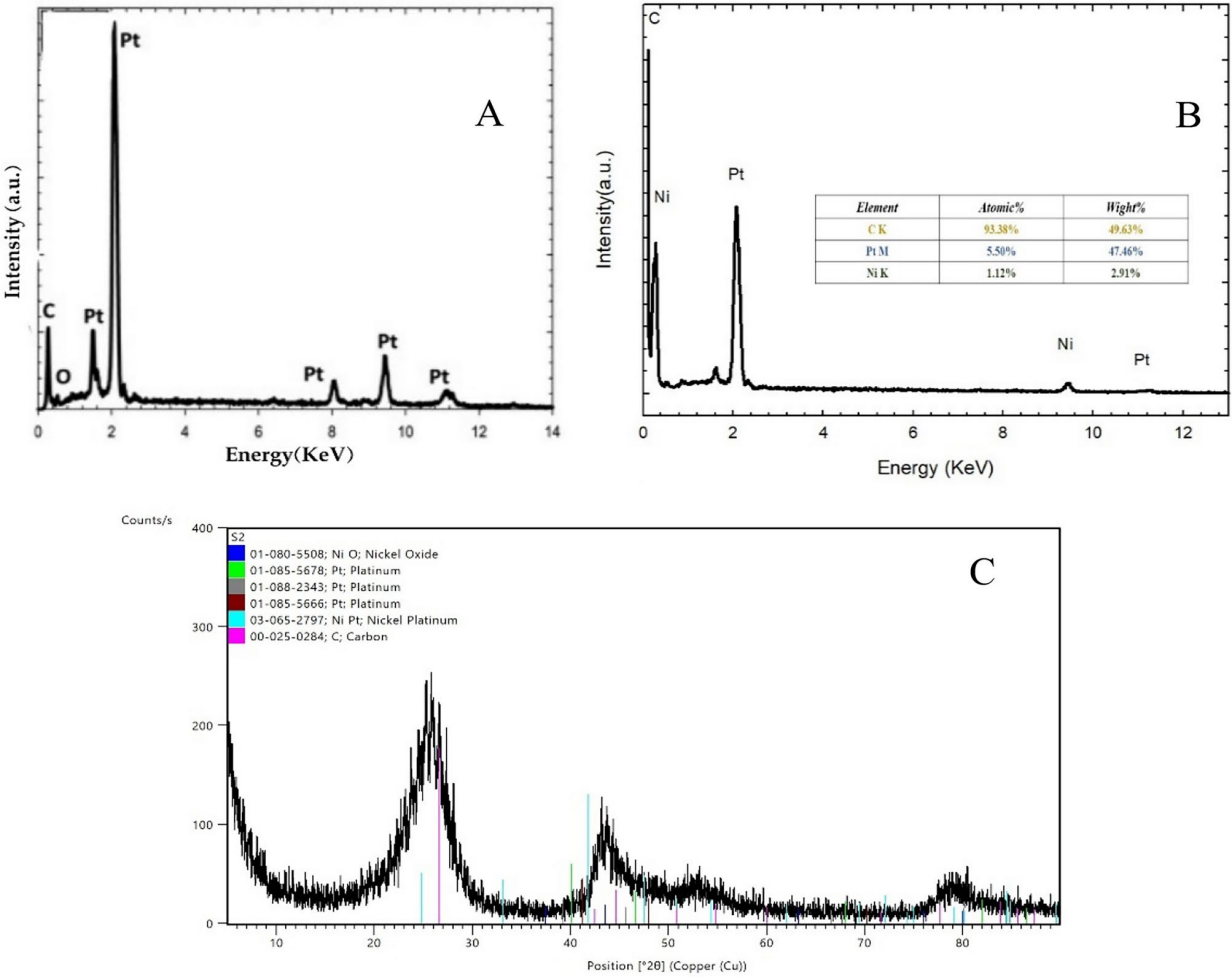
EDX spectra of (**A**) Pt/GC, (**B**) NiOx/Pt/GC. (**C**) XRD spectrum of NiOx/Pt/GC electrodes.

The corresponding EDX spectral analysis are shown in Fig. 3, which indicates evidence for the successful electrodeposition of nano-Pt onto the surface of GC (A) while, image (B) shows the presence of both Pt and Ni on GC surface. XRD spectrum Fig. 3C reprecents the precence three main characteristic peaks of Pt and Ni at 2θ=25, 43° and 78° (COD 03-065-2797). Also, characteristic peaks of carbon of GC electrod are also present acording to (COD 00-025-0284)^21^.

**Fig. 4:**
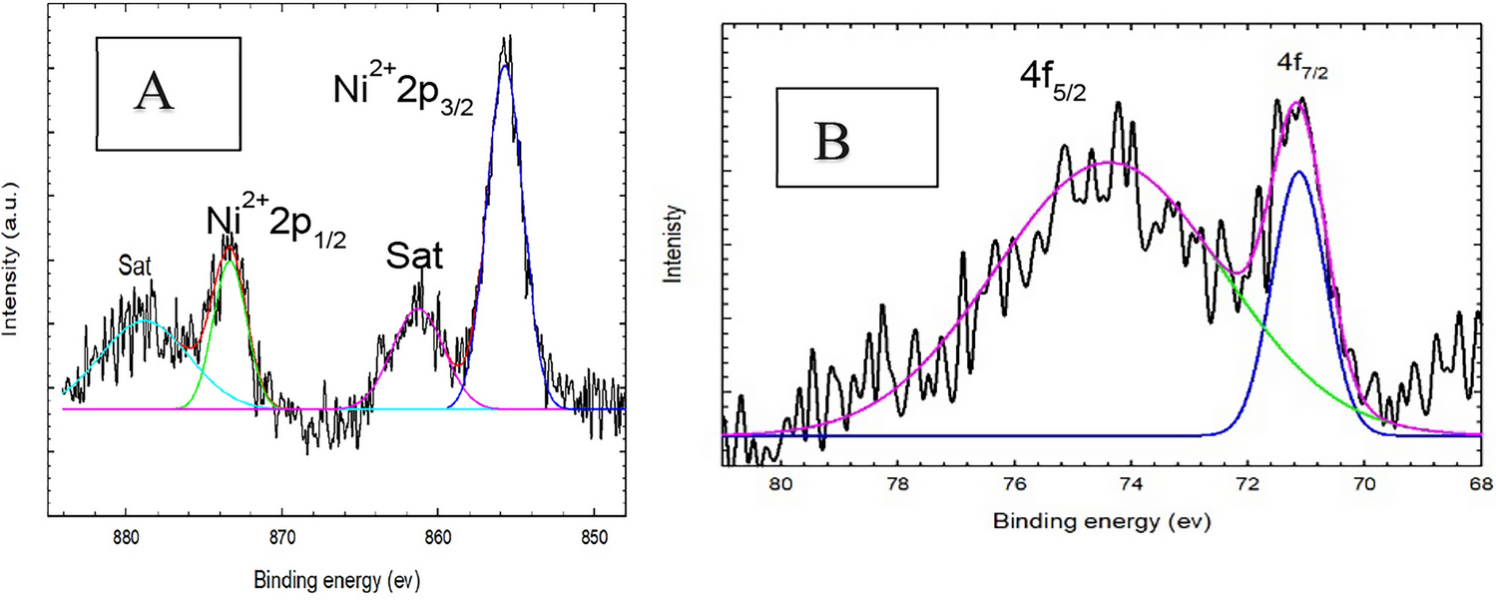
XPS spectra of (**A**) Ni2p and (**B**) Pt4f of NiOx/Pt/GC electrode.

The corresponding XPS spectra of Ni 2p and Pt 4f (Fig. 4A and B) of the as-prepared NiOx/Pt/GC catalysts. It shows clear characteristics peaks for Ni^+2^ due to surface oxidation at 855 and 873 eV, and Pt shows 4f_7/2_ and 4f_5/2_ peaks at ca. 71 and ca. 75 eV, respectively, corresponding to the presence of elemental Pt^22^.

**Fig. 5:**
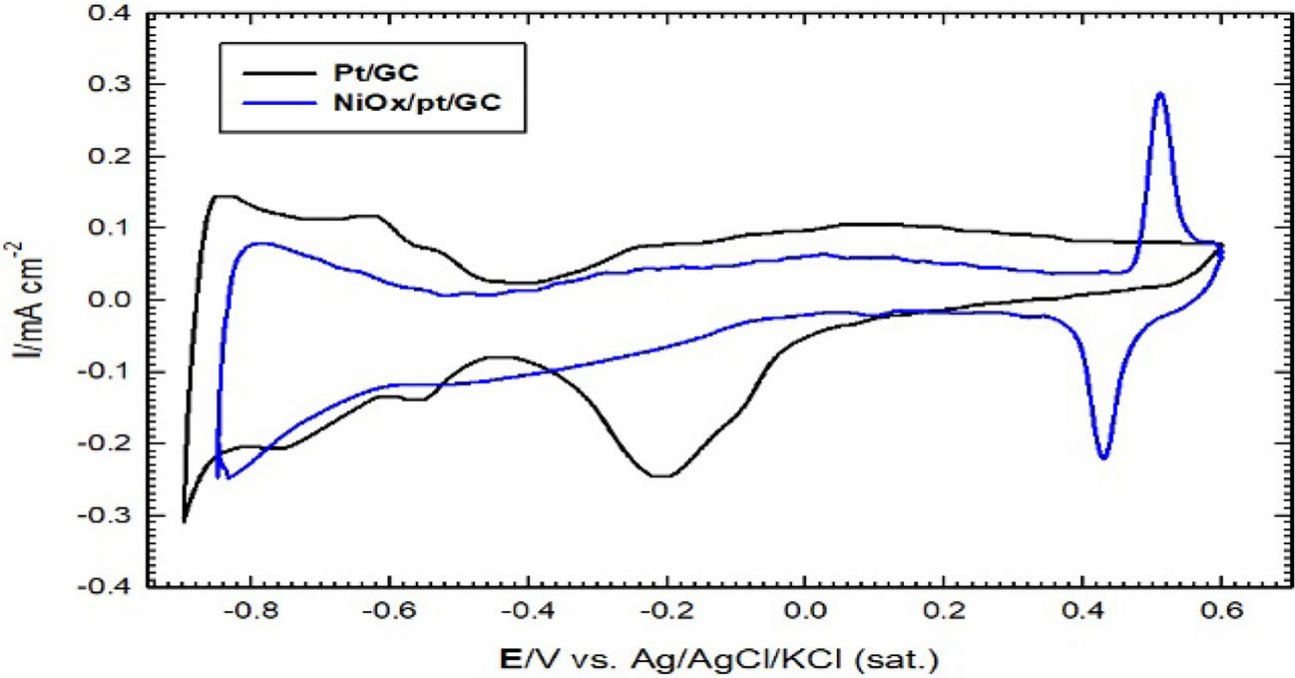
CV of Pt/GC (black line) and nano-NiOx/Pt/GC (blue line) measured in 0.1 M KOH. Potential scan rate: 50 mV/s.

A typical characteristic CV (Fig. 5) for a Pt substrate is observed in 0.1 M KOH at nano-Pt/GC electrode in which a broad reduction peak (at ca. − 0.26 V) for the Pt in addition to hydrogen adsorption/desorption (H_ads/des_) couple appeared in the potential region from − 0.6 to − 0.9 V for the nano-Pt/GC electrode (line in black color).When NiOx is electrodeposited, a noticeable decrease in the intensity of the reduction peak of the Pt oxide (which commences at ca. − 0.26 V) is observed along with a decrease in the current of the H_ads/des_ peaks (in the potential region from − 0.5 to − 0.85 V vs. Ag/AgCl/KCl (sat)). Whereas, a redox couple peak is observed, at ca. 0.45 to 0.5 V, which corresponds to the redox transformation of Ni(OH)_2_/NiOOH^22^. It is worthy to mention here that the electrochemical active surface area of the nano-Pt/GC and nano-NiOx/Pt/GC was estimated by probing the features of the characteristic CV response measured in 0.1 M KOH (Fig. 5, in which the Pt-oxide formation commences at ca. − 0.26 V till 0.6 V, and the corresponding reduction of the Pt-oxide is observed during the negative-going potential scan at ca. − 0.2 V. The charge of the reduction peak of Pt-oxide was taken to estimate the real surface area of Pt nanoparticles using a reported value of 420 micro C/cm^2^. The intensity of this peak is much decreased upon the further modification of Pt/GC with nano-NiOx. This indicates the partial coverage of Pt with NiOx as evident by comparing the two CV diagrams shown in Fig. 5.

### Electro-oxidation of FA

#### Urea as a blending fuel

**Fig. 6:**
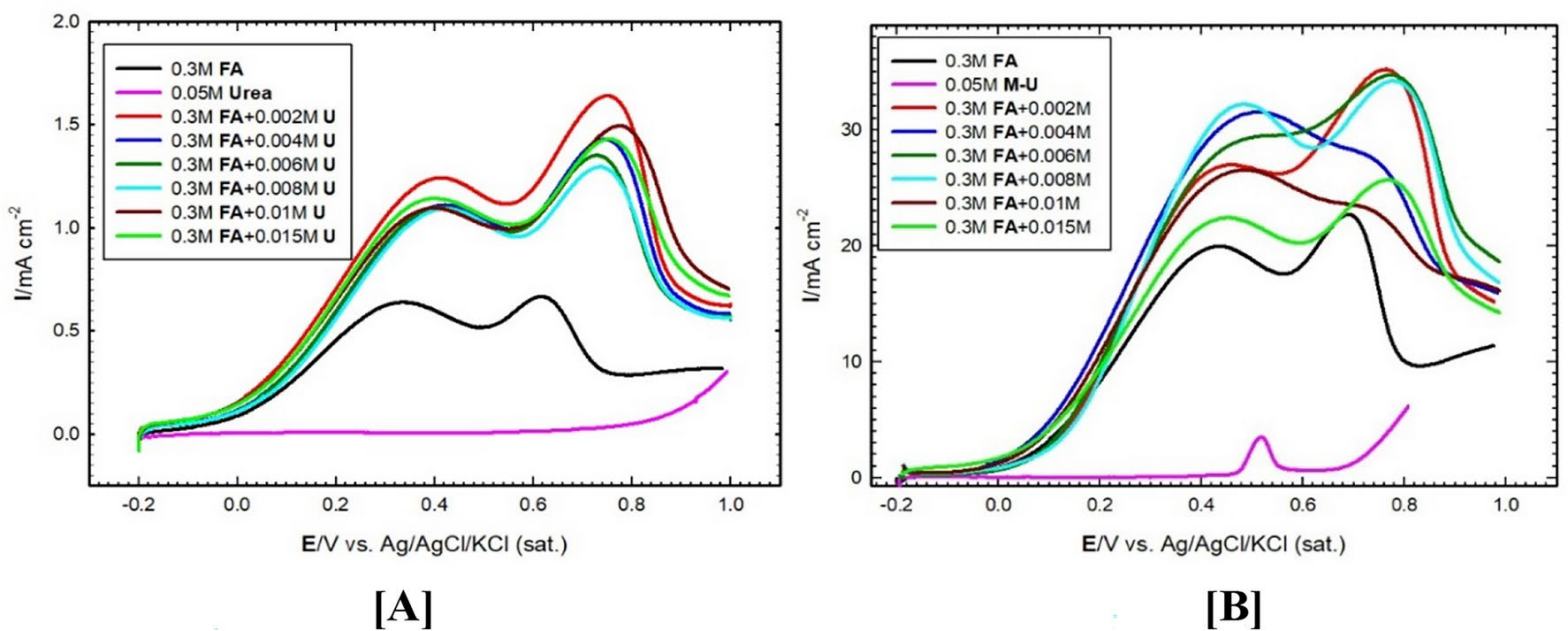
LSV of FAO at (**A**) Pt/GC and (**B**) NiOx/Pt/GC electrodes from 0.1 M KOH containing + 0.3 M FA (pH = 3.5) in the absence (black line) and the presence of various concentrations of urea (colored lines). Potential scan rate = 50 mV/s. N.B. the potential scan starts at − 0.2 V towards the positive-going potential direction.

Herein, with FA concentration 0.3 M and (pH = 3.5) electrooxidation at Pt/GC (Fig. 6A) and at NiOx/Pt/GC (Fig. 6B) electrodes (black curves in Fig. 6) shows two peaks the first one for direct oxidation of FA to CO_2_ with current density (*I*_p_^d^), while the second peak stands for indirect oxidation of FA, (i.e., oxidation of CO produced from the non-faradaic dissociation of FA) to CO_2_ with a current density of (*I*_p_^ind^). By adding urea (U) as a blending fuel (with various concentrations), the onset potential of FAO shifts to more negative and current density increased for direct and slightly decreases for the indirect oxidation of FA which indicates that U enhances the oxidation of FA.

**Fig. 7:**
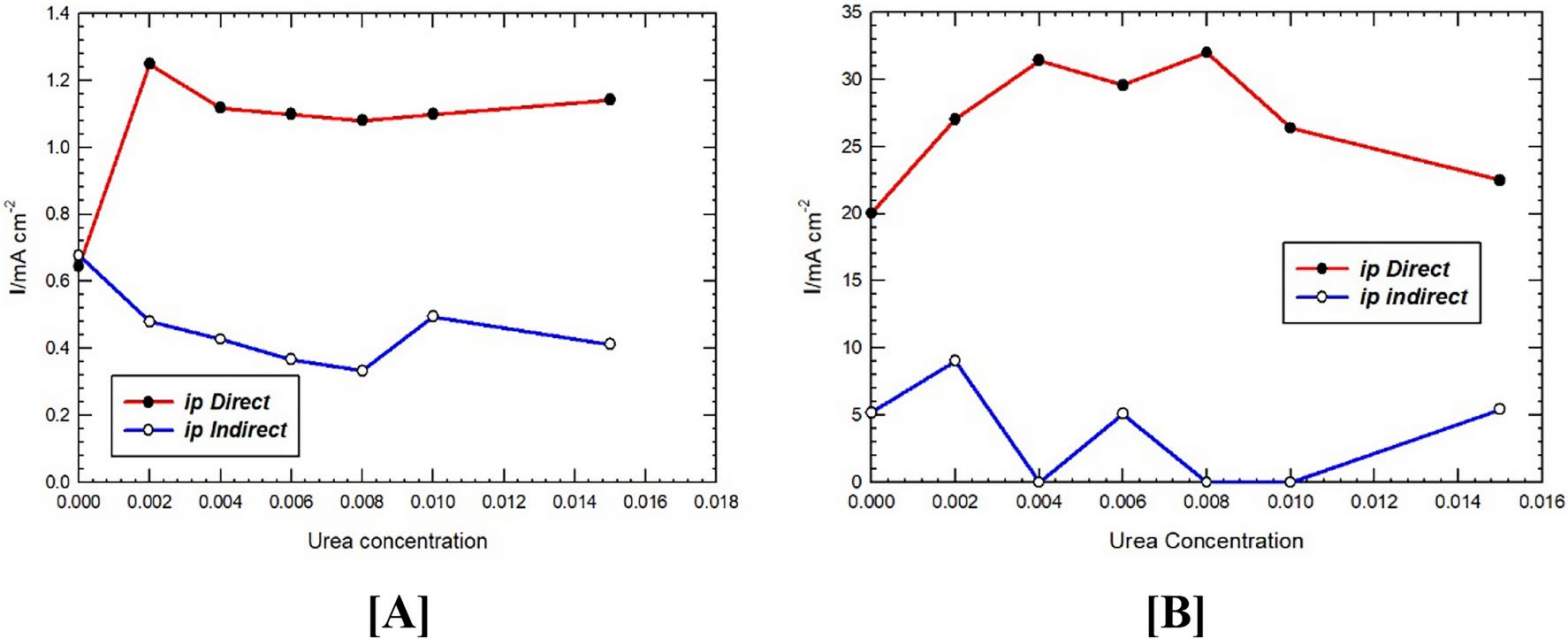
Variation of *I*_p_-direct and *I*_p_-indirect for FAO at (**A**) Pt/GC and (**B**) NiOx/Pt/GC electrodes vs. urea added. Data were abstracted from Fig. 6.

Figure 7 shows the relation between *I*_p_^d^ and *I*_p_^ind^ for FAO in the presence of various additions of U. It is clear that U increases the direct oxidation current of FA albeit to various extents, and concurrently, decreases the indirect oxidation pathways. This could be attributed to the change of the dipole moment of FA compared with the binary blend of FA-U formed via H-bonding (c.f. DFT calculations section). This results in a favorable adsorption geometry of FA which assisted in the C-H bond cleavage of FA to form CO_2_.

A significant change in both direct and indirect currents of FAO has been observed. The obvious increase of *I*_p_^d^ is noticed by the addition of 2 mM urea (Fig. 7A). Further increase of U, did not show any significant enhancement of the direct FAO. Similarly, *I*_p_^d^ is enhanced by addition of minute amounts of urea with minimal values of *I*_p_^ind^ in the presence of 4 mM U as depicted from Fig. 7B.

#### Urea derivatives as blending fuels

**Fig. 8:**
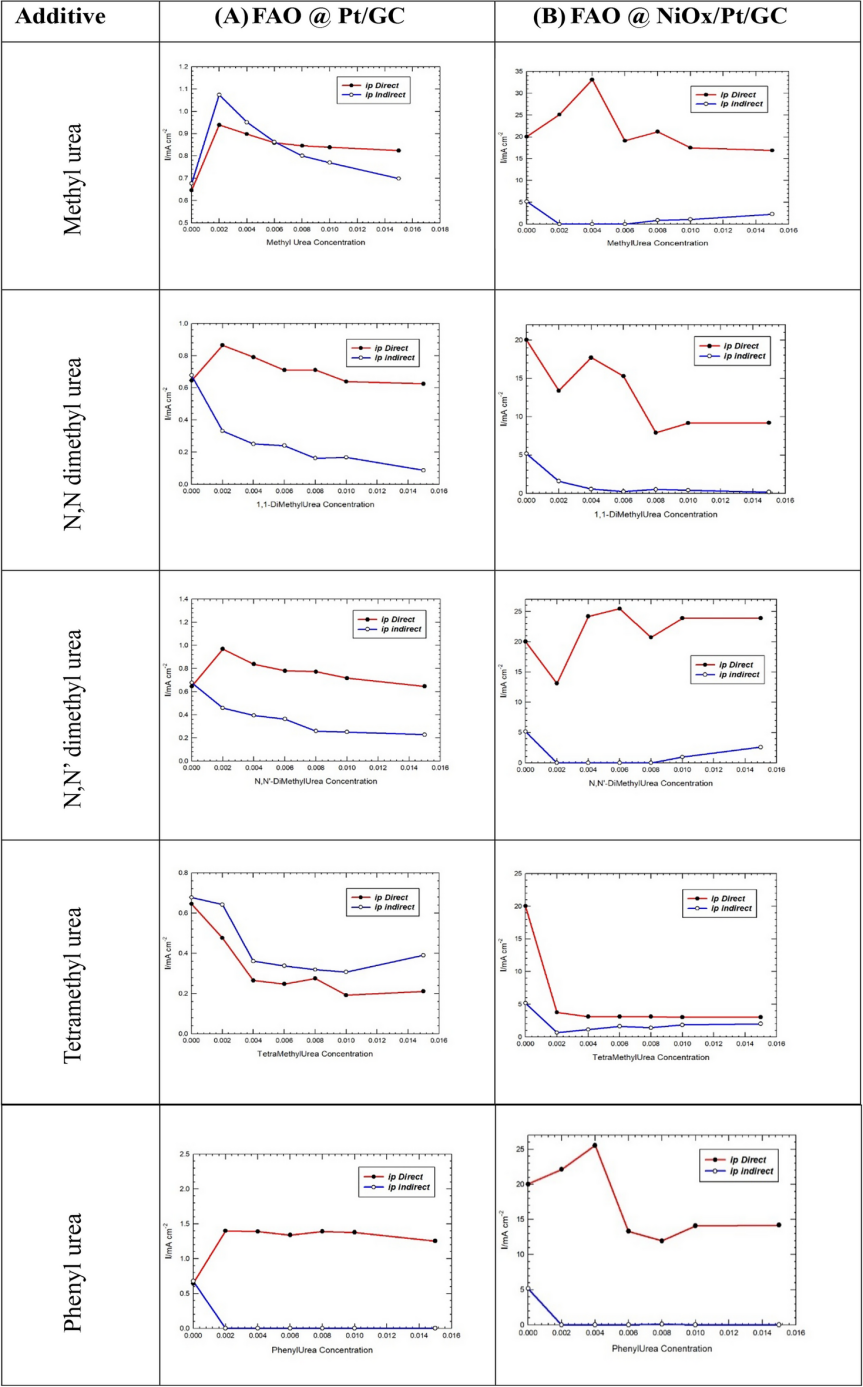
Variation of *I*_p_^d^ and *I*_p_^ind^ for FAO at (**A**) Pt/GC and (**B**) NiOx/Pt/GC electrodes vs. urea derivatives added.

Herein, the impact of urea derivatives is investigated by replacement of hydrogen atom(s) of the –NH_2_ group of U by methyl groups or phenyl group. Thus, four derivatives were investigated, i.e., methyl urea, dimethyl urea (with either the two methyl at the same N atom or each methyl at each N atom), tetramethyl urea and phenyl urea. The behavior of FA oxidation in presence of various concentrations of each additive is investigated by measuring LSVs (similar to those shown in Fig. 6 above) and similar data were abstracted and presented in Fig. 8 for the variation of *I*_p_^d^ and *I*_p_^ind^ for FAO for each case (similar to the data presented in Fig. 7).

Inspection of Fig. 8 reveals the following points:

1. Methyl urea (MU) on FAO at nano-NiOx/Pt/GC shows a significant increase in *I*_p_^d^ is observed with the addition of a minute amounts of MU up to 4 mM, beyond which a decrease in *I*_p_^d^ in noticed due to the blocking effect of Pt active sites by MU.
2. Surprisingly, *I*_p_^d^ is reduced with a simultaneous retardation of *I*_p_^ind^ upon the addition of N,N dimethyl urea ( a derivative with two methyl groups at the same nitrogen atom). On the contrary, the addition of N,N′ dimethyl urea derivative (with one methyl group at each nitrogen atom) shows a significant enhancing effect of *I*_p_^d^ of FA. This observation highlights that the two terminal H atoms at the two nitrogen atoms play a significant role possibly in favoring a certain orientation of FA in the vicinity of the electrode surface such that facilitating the C-H bond cleave. A plausible explanation might suggest the formation of 8-membered ring within the double layer region between FA and urea derivative with two terminal H atoms at the two nitrogen atoms, thus directing FA properly at the electrode surface. The absence of at least one H atom at each nitrogen disallow the formation of such a binary compound, but causes a retarding effect towards FAO as observed in the case of N,N dimethyl urea.
3. To further support the above assumption, tetra-methyl urea (with no H atoms at either N) shows a negative impact on FAO by reducing both *I*_p_^d^ as well as *I*_p_^ind^ at both Pt/GC and nano-NiOx/Pt/GC electrodes. This could be reasonably attributed to the inability of tetramethyl urea to forvorably oriente FA at the electrode surface (due to the absence of H atoms at the two terminal N atoms) as well as its bulky nature which is believed to block the Pt active sites thus retarding the overall FAO process.
4. For the good fortune, phenyl urea (PU) shows the best enhancement towards the direct FAO, with almost no *I*_p_^ind^. This is reflected in a marked shift of the onset potential as well of FAO (by ca. 150 mV towards the negative direction, LSV data are not shown). The superb enhancing effect of PU over U and/or its derivatives (investigated herein) is attributed mainly to the possible formation of the 8-membered ring (due to the presence of at least one H atom at each N atom) together with the inductive effect of the phenyl group attached to urea, which enhances the dipole moment of the binary FA-PU fuel blend and induces a marked stability as well (c.f. the DFT calculations section). Noteworthy mentioning here *I*_p_^ind^ diminished at the two electrodes over the employed concentration range of PU. This means that PU forces FA to be directly oxidized to CO_2_.

### Comparison and DFT calculations

DFT calculations (shown in Table 1) support the experimental work as experimental work showed that phenyl urea has highest enhancement of FA electrooxidation as this blend has most stabilized energy and high polarity which made it easier for FA to be attracted to catalyst layer due to high electron density on the phenyl group in phenyl urea. Also (PU) has highest positive *E*_HOMO_ value which means it has high tendency for electron donating character. Inspection of this table reveals the higher stability of the binary fuels compared to the individual fuels. The dipole moment is an important parameter which affects the favorable adsorption geometry of the respective fuel onto the catalyst surface. The nominal stability of PU compared to others together with its dipole moment promotes the direct oxidation of FA to CO_2_.

## Conclusion

This study investigated the impact of urea derivatives on the preferential oxidation pathway of FA (either the direct pathway to CO_2_, or the indirection oxidation pathway via CO intermediate formation). That is the use of mono- and di-methyl urea derivatives (i.e., with progressive substitution of H with methyl groups) as blending fuels showed higher direct oxidation current of FA, at both Pt/GC and NiOx/Pt/GC electrodes, albeit to lower extents than urea. Conversely, tetramethyl urea (TMU, as a blending fuel to FA) showed a retarding effect on the direct oxidation current of FA. This could be explained in view of the bulky nature of the TMU which blocked the Pt active sites. Surprisingly, phenyl urea (PU, as a blending fuel with FA) showed the highest direct oxidation current in the presence of 2.0 mM PU + 0.3 M FA. Further additions of PU to FA did not improve the FA oxidation anymore, where a plateau is obtained. This could be explained in view of the withdrawal character of the phenyl group which strengthen the H-bonding between FA and PU, thus stabilizing the binary fuel blend, with higher dipole moment than urea or FA alone. DFT calculations supported the proposed interaction of FA with the various urea derivatives, in which the calculated dipole moments of the binary fuels was in-line with the observed enhancement in the electro-oxidation of FA.

## Data Availability

The datasets used and/or analysed during the current study available from the corresponding author on reasonable request.
