# Author Correction: Optimization and DFT study for boosted electooxidation of formic acid at NiOx modified Pt using urea derivatives as blending fuels

**DOI:** 10.1038/s41598-025-97454-w

**Published:** 2025-04-09

**Authors:** Aya M. Saada, Mohamed E. Ghaith, Ahmed A. El-Sherif, Mohamed S. El-Deab

**Affiliations:** 1 Department of Biochemical Engineering, Faculty of Energy and Environmental Engineering (FEEE), The British University in Egypt (BUE), El-Shorouk, Cairo 11837 Egypt; 2 Department of Chemistry, Faculty of Science, Cairo University, Cairo, Egypt

Correction to: *Scientific Reports* 10.1038/s41598-024-84492-z, published online 18 January 2025

The original version of this Article contained an error in the spelling of the author Mohamed E. Ghaith which was incorrectly given as Mohamed E. Gaith.

The original Article has been corrected.

